# Gender-based differences in the relationships among proactive personality, perceived entrepreneurial support and entrepreneurial intention of Chinese private college students: A moderated mediation model

**DOI:** 10.3389/fpsyg.2022.871343

**Published:** 2022-08-25

**Authors:** Jing Tian, Mohan Zhang, Yunpeng Wu, Haitao Zhou

**Affiliations:** ^1^Zhejiang Academy of Higher Education, Hangzhou Dianzi University, Hangzhou, China; ^2^Jing Hengyi School of Education, Hangzhou Normal University, Hangzhou, China; ^3^School of Teacher Education, Dezhou University, Dezhou, China; ^4^Faculty of Education, Beijing Normal University, Beijing, China

**Keywords:** proactive personality, entrepreneurial intention, perceived entrepreneurial support, gender, Chinese private college

## Abstract

Proactive personality is a key determinant of entrepreneurial intention. Few studies have explored the mediating and moderating mechanisms underlying this relationship. This study investigates the association between proactive personality and entrepreneurial intention and examines the mediating role of perceived entrepreneurial support and the moderating role of gender. Using a cross-sectional design, 1,515 senior students (women = 838, men = 677) from Chinese private colleges were recruited using random cluster sampling. They completed a battery of self-reported online questionnaires on proactive personality, perceived entrepreneurial support, and entrepreneurial intention. The results revealed that perceived entrepreneurial support mediates the association between proactive personality and entrepreneurial intention. Moderated mediation analysis indicated that the relationship between proactive personality and perceived entrepreneurial support and that between perceived entrepreneurial support and entrepreneurial intention are moderated by gender. Specifically, the positive association between proactive personality and entrepreneurial intention was stronger in male students, and the positive association between perceived entrepreneurial support and entrepreneurial intention was stronger in female students. This study contributes to the understanding of how proactive personality predicts entrepreneurial intention in Chinese private college students and bears implications for higher education institutions and policymakers that entrepreneurship promotion agenda should focus on improving perceived entrepreneurial support and considering the gender of students.

## Introduction

Entrepreneurship and innovation have become mainstream of the economic and social reforms with the momentum of economic development and prosperity. In recent years, entrepreneurship promotion initiatives have boomed locally and globally. In 2015, Chinese government issued a nationwide entrepreneurship and innovation policy to stimulate mass creativity, increase employment, and promote vertical social mobility and social equality. Higher education institutions act as the engines of socioeconomic development, and college students are the driving force of entrepreneurship. To stimulate the entrepreneurial vitality and obtain fuller and high-quality employment for college students, Chinese state council introduced the Guidance on Further Supporting Innovation and Entrepreneurship of College Students in 2021, which placed great emphasis on building the entrepreneurship and innovative-oriented college students training model and optimizing college students’ entrepreneurship environment. Higher education institutions at all levels in China have responded to the policies by setting up entrepreneurial education courses and providing institutional entrepreneurship support.

Entrepreneurial intention refers to the self-acknowledged conviction and preparation for establishing a new business venture, adding value to an existing organization, or consciously planning to do so in the future (Wang et al., 2016). It has received considerable attention for positively influencing entrepreneurial behavior. In terms of entrepreneurial intention among college students, there is a negative relationship between the hierarchy of higher education institutions and entrepreneurial intention in China (Zhong et al., 2016). Accordingly, students in Chinese private colleges have stronger entrepreneurial intention because most private colleges are in the middle and lower layers of the hierarchical structure of Chinese higher education institutions. Although entrepreneurial intention is gaining increasing prominence, most studies on the entrepreneurial intention of Chinese higher education institutions focus on public colleges, which are prestigious and research-oriented higher education institutions. With the application orientation, Chinese private colleges can be keenly aware of market demand and take advantages of the combination of colleges and enterprises, which has not received sufficient attention in current entrepreneurship studies. Previous studies revealed Chinese college students’ entrepreneurial intention and the predicted factors, such as attitude, personality, self-efficacy, sociodemographic exposure to supportive environment, and so on (Yang, 2013; Hu et al., 2019; Liu et al., 2019). Specifically, proactive personality traits and entrepreneurial support are prerequisites for entrepreneurship (Crant, 1996; Neneh, 2019a). However, how they contribute to college students’ entrepreneurial intention requires further investigation. This study aims to investigate Chinese private college students’ entrepreneurial intention, its key impacting factors, and the mechanism, and it is worthy to obtain additional evidence to remedy the deficiencies in previous studies.

## Literature review and hypothesis development

### Proactive personality and entrepreneurial intention

Personality is an individual’s cognitive, affective, conative, and physical characteristics, and it describes and differentiates various manifestations and nuances of different people (Eysenck, 1946). Personality traits, such as extraversion, neutralism, optimism, risk-taking propensity, achievement orientation, tolerance for ambiguity, interpersonal relationship, internal locus of control, creativity, empathy, and moral obligations, have been widely discussed and proven to be positively associated with entrepreneurial intention (Munir et al., 2019; Bergner et al., 2021). To evaluate the contribution of various personalities to understanding entrepreneurship intention, the focus has shifted from broad personality traits to narrow ones. Regarding positive psychological attributes, proactive personality demonstrates a positive explanatory power in entrepreneurial intention (Baluku et al., 2020). Proactive personality is not only a belief in a person’s ability to overcome situational challenges but also can enhance creativity (Neneh, 2019a), and people with proactive personality tend to take the initiative to create new opportunities or improve their current circumstances, rather than passively adapt to the conditions when confronting difficulties. Proactive personality has a robust relationship with entrepreneurs as entrepreneurship is an innovative activity that needs endogenous power (Crant, 2000). Therefore, proactive personality has long been seen as a crucial predictor of entrepreneurial intention because entrepreneurship relies heavily on individual initiatives.

Contextual and individual factors had significant relationship with entrepreneurial intention, and proactive personality, as individual factors, positively and significantly moderated the relationship between entrepreneurial intention and entrepreneurial behavior (Salami, 2019; Li et al., 2020). According to Paul et al. (2017), a country’s culture and an individual’s proactive personality are the most important antecedents of entrepreneurial intention. Proactive personality implies initiative, adaptability and creativity, whereas traditional Chinese culture values are modest, discreet, and introverted. Moreover, students’ initiative and agents in Chinese private colleges are usually inferior to their public peers from the perspective of enrollment quality and academic performance. Considering the uniqueness of Chinese private college students and consistent with the above literature, our first hypothesis focuses on replicating these relationships in the context of Chinese private college.

**Hypothesis 1:** Proactive personality is positively associated with entrepreneurial intention.

### Mediating effect of perceived entrepreneurial support

Entrepreneurship is a highly resource-dependent activity, and the extent of the availability of capital resources and access to policy support are significant enhancers of entrepreneurial intention. The impacts of various types of entrepreneurial support, such as parental socioeconomic status, financial support, network, institutional, and university support on entrepreneurial intentions and behaviors are noted. Yi (2021) found that external institutional support is the key intermediary variable that plays important role in turning entrepreneurial intention into entrepreneurial behavior. Perceived entrepreneurial support refers to individuals’ attitude and value toward contextual and situational entrepreneurial resources, and there is a positive relationship between personal perceptions with respect to the supportiveness of a given society and institution, the business environment (Díaz-García and Jiménez-Moreno, 2010), and entrepreneurial intentions (Shi et al., 2019).

Entrepreneurial support has a significant influence on entrepreneurial attitude, and this impact is regulated by personal characteristics (Huang et al., 2020). In terms of emphasizing individual initiative of making use of resources and taking actions to reach the goals (Bateman and Crant, 1993), proactive personality is closely related to perceived college and external entrepreneurial support in entrepreneurship. Although it is widely known that college support and the exogenous environment are the dominant socioeconomic milieu shaping college students’ entrepreneurial intention, they have an indirect impact on shaping of entrepreneurial intention among students (Trivedi, 2017). Previous studies have examined the mediating roles of entrepreneurial alertness (Hu et al., 2018; Neneh, 2019b), entrepreneurial self-efficacy (Prabhu et al., 2012), and the components of the theory of planned behavior (Ana et al., 2019) on the relationship between proactive personality and entrepreneurial intention and how perceived entrepreneurial support mediates proactive personality and entrepreneurial intention. With a growing number of Chinese private colleges embracing the notion of mass entrepreneurship, they have constructed entrepreneurial support systems. However, the role of a college as a provider and enabler of entrepreneurial support and its impact on entrepreneurial intention in students has not been studied in Chinese private college. We can possibly extend the previously mentioned relationships and findings from the studies of perceived entrepreneurial support to our second hypothesis.

**Hypothesis 2**: Perceived entrepreneurial support mediates the relationship between proactive personality and entrepreneurial intention.

### Mediating effect of gender

Based on social role theory, previous studies revealed the gender differences on entrepreneurial intention, men were perceived to have superior pro-entrepreneurial personality traits than women, with various type of disadvantages caused by their socially prescribed gender, women displayed lower entrepreneurial intentions than men (Bagheri and Lope Pihie, 2014; Villanueva-Flores et al., 2021). In line with these findings, some variables associated with gender, which determines entrepreneurial intention, were also investigated. For instance, entrepreneurship education and entrepreneurial attributes include risk management, creativity, problem management, subjective norm, and psychological capital (Díaz-García and Jiménez-Moreno, 2010; Abbas, 2015; Nowiński et al., 2019). Based on an investigation of the personality traits of risk tolerance, optimism, achievement orientation, and creativity, Roy and Das (2020) found that gender acts as a moderator between personality traits and entrepreneurial intention in India, where female entrepreneurship suffers significantly when set up a business. With external support being important in the study of gender and entrepreneurial intention, Sidratulmunthah et al. (2018) recognized that the relationship between support perception and entrepreneurial intention was moderated by gender. Although the impact of personality traits on entrepreneurial intention has been frequently discussed, few studies have empirically examined how proactive personality, one of the most important instinct personality traits, particularly along with the mediating role of perceived entrepreneurial support, affects entrepreneurial intention among college students.

Many studies have assessed gender differences in the formation of college students’ entrepreneurial intention. However, the findings are inconsistent. While the prevailing arguments claim that entrepreneurship is generally regarded as a field for men, women tend not to choose entrepreneurship because of gender stereotypes, and gender significantly moderates the relationship between students’ entrepreneurial intention and its antecedents (Yang, 2013; Bagheri and Lope Pihie, 2014). Others have concluded that there is no statistical difference in entrepreneurial intention between male and female populations in certain contexts (Abbas, 2015; Contreras-Barraza et al., 2021). Specifically, based on the empirical study of 462 students from Chinese public and private higher vocational colleges, Li and Islam (2021) claimed that gender did not significantly moderate the relationship between entrepreneurial education and entrepreneurial policy on entrepreneurial self-efficacy and intention. The interaction between gender and entrepreneurial support factors are not fully studied to understand entrepreneurial intention among college students in developing countries (Rahaman et al., 2020). The role of gender in entrepreneurial intention that can be a fertile place in times of importance for female workers in the economy has been emphasized. As such, to better understand the personality-entrepreneurial intentions link, this study introduced an important moderator (i.e., gender) that could be instrumental in explaining why some individuals have strong intentions while others do not. This study expected that gender would affect the relationships among proactive personality, perceived entrepreneurial support, and entrepreneurial intention. Therefore, we proposed the following hypotheses:

**Hypothesis 3:** Gender moderates the relationship between proactive personality and entrepreneurial intention.

**Hypothesis 4:** Gender moderates the relationship between proactive personality and perceived entrepreneurial support.

**Hypothesis 5:** Gender moderates the relationship between perceived entrepreneurial support and entrepreneurial intention.

### Current study

Previous studies provide an ambiguous picture in respect to the impact of perceived entrepreneurial support and gender on the link between personality and entrepreneurial intention. To develop a framework of entrepreneurial intention, this study aimed to corroborate how the major determinants of the entrepreneurship intention outline students’ entrepreneurship in Chinese private college. To provide a broader scenario, this study investigated individual (proactive personality) and environmental context (perceived entrepreneurial support) that may influence entrepreneurial intention. In addition, the potential moderating role of gender was investigated. For practical implication, this study considered the need to inspect the core factor while attaining a superior understanding of Chinese private college students’ intention to develop career through entrepreneurship and, additionally, interplaying the influence of gender on these factors.

**FIGURE 1:**
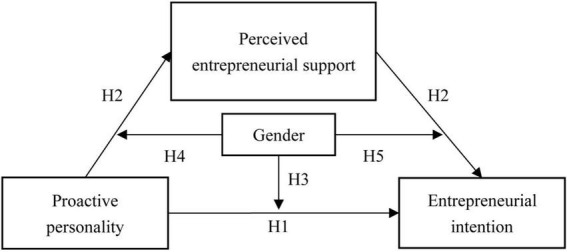
Conceptual model.

This study examined the relationship between proactive personality and entrepreneurial intention and its underlying mechanism. We proposed a moderated mediation model (Figure 1) and attempted to answer (a) whether perceived entrepreneurial support mediates the association between proactive personality and entrepreneurial intention and (b) whether gender moderates each path of medication process.

## Materials and methods

### Participants and procedures

This study adopted data from the 2016 Chinese Private College Students Survey (CPCSS) conducted by Private Education Research Team of Beijing Normal University. The random cluster sampling method was used. Data were collected through online questionnaires on the Wenjuanxing platform. All participants provided informed consent in advance and were asked to complete an anonymous self-reported questionnaire, which took 15–20 mins. The total number of the sampling was 58,710. Among all the valid samplings, 1,515 senior students from private colleges in Jiangsu, Henan, Sichuan, Heilongjiang, and several other provinces were included in this study, including 677 (44.7%) men, 838 (55.3%) women, 1,115 (73.6%) students aged above 22 years, and 400 (26.4%) students aged below 22 years (Table 1).

**TABLE 1:** Demographic statistics (*N* = 1,515).

Variables	Frequency (*n*)	Percent (%)
**Gender**		
Female	838	55
Male	677	45
**Age group**		
18 years and below	25	2
19 years	35	2
20 years	74	5
21 years	266	18
22 years and above	1,115	73
**Province**		
Jiangsu	240	16
Henan	831	55
Heilongjiang	227	15
Sichuan	161	11
Other provinces	56	3

### Measures

#### Proactive personality

The proactive people tend to have strong desire to succeed when they are determined to set up a business, and a proactive personality is crucial in bringing entrepreneurial intention to fruition. Typically, individuals with a proactive personality are action-oriented and can provide innovative ideas and make changes in the environment through continuous learning and improvement, relying on their initiatives (Paul et al., 2017). The questionnaire measures college students’ personal disposition toward proactive behavior by employing six items from prior studies (Crant, 1995; Paul et al., 2017; Neneh, 2019a). The items were as follows: (1) I believe some personality traits are changeable, (2) I can work autonomously without supervision, (3) I can actively deal with the challenge of new environment, (4) I can work effectively under pressure, (5) I can take conscientious action, and (6) I can learn continuously. Each item was rated on a five-point Likert-type scale ranging from 1 (strongly disagree) to 5 (strongly agree). In this study, the Cronbach’s α was 0.951.

#### Perceived entrepreneurial support

Studies claim a relationship between economic and entrepreneurial activities. Although students with high socioeconomic background have stronger entrepreneurial intention than students with low socioeconomic background (Mosey et al., 2012; Castaño et al., 2016), access to finance is not sufficient to influence entrepreneurial intention, unless combined with entrepreneurial behavioral control (Nguyen, 2020). Entrepreneurial intentions are related to personal perceptions with respect to the supportiveness of a given society (Díaz-García and Jiménez-Moreno, 2010). Ghosh (2017) indicated that public policy is crucial factor that determines college students’ entrepreneurial intention. Therefore, much emphasis was placed on students’ subjective perceptions about the policy and college support when measuring perceived entrepreneurial support in this study. The following five items were applied: (1) It is easy for me to obtain the start-up capital, (2) It is easy for me to access entrepreneurship rules and regulations, (3) There are favorable entrepreneurship policies available to me, (4) Supportive entrepreneurship rules and regulations exist, and (5) I can access adequate entrepreneurship guidance and support services at my college. Participants answered the items on a five-point scale from 1 (strongly disagree) to 5 (strongly agree). High scores indicate higher level of perceived entrepreneurial support. Cronbach’s α was 0.940 in this study.

#### Entrepreneurial intention

Entrepreneurial intention refers to individuals’ subjective attitude toward dedicating themselves to entrepreneurial activities, which is ascribed to potential or successful entrepreneurs. To measure the entrepreneurial intention construct, we adopted 2 items focusing on the key dimensions: (1) I have entrepreneurial intention to start up a firm independently and (2) I have clear plan to set up a business. The questions were measured on a five-point Likert scale from 1 (strongly disagree) to 5 (strongly agree), with higher score indicating higher level of entrepreneurial intention. Cronbach’s α for entrepreneurial intention measurement was 0.902 in this study.

### Statistical analysis

Data analysis were performed using SPSS 22.0 (IBM, Chicago, IL, United States). First, descriptive statistics and Pearson correlation analysis were conducted. Second, the mediating effect of perceived entrepreneurial support (hypotheses 1 and 2) was tested using Hayes (2013) SPSS macro-PROCESS (Model 4). Third, the moderating effect of gender (hypotheses 3, 4, and 5) was tested using SPSS macro-PROCESS (Model 59). The bootstrapping method with robust standard errors was applied to test the significance of the effects (Hayes, 2013). It produced 95% bias-corrected confidence intervals (CIs) of these effects from 5,000 resamples of the data. If CIs did not include zero, the effects in Models 4 and 59 were significant at α = 0.05. As gender is a dichotomous variable, simple slopes were plotted using its categorical level (men and women).

## Results

### Preliminary analysis

Means, standard deviations (SDs), and Pearson’s correlation coefficients for the key variables were examined (Table 2). The correlations between the variables of interest were significant and in the expected directions. Proactive personality was positively associated with perceived entrepreneurial support and intention, and the perceived entrepreneurial support was positively correlated with entrepreneurial intention.

**TABLE 2:** Means, standard deviations, and Pearson correlation coefficients for key variables (*N* = 1,515).

	*M*	*SD*	1	2	3
1. Proactive personality	4.04	0.79	–		
2. Perceived entrepreneurial support	3.75	0.91	0.678***	–	
3. Entrepreneurial intention	3.86	0.90	0.733***	0.773***	–

***p < 0.001.

### Testing for the mediation model

We conducted a PROCESS macro for SPSS (Model 4) to examine hypotheses 1 and 2. Table 3 shows a significant total effect of proactive personality on entrepreneurial intention [total effect = 0.841, SE = 0.20, 95% CI = (0.80, 0.88)]. Thus, hypothesis 1 is supported. Hypothesis 2 predicted that perceived entrepreneurial support mediates the relationship between proactive personality and entrepreneurial intention. Table 3 shows that proactive personality is positively linked with perceived entrepreneurial support (β = 0.788, *p* < 0.001), which, in turn, is positively associated with entrepreneurial intention (β = 0.503, *p* < 0.001). The positive direct association between proactive personality and entrepreneurial intention remained significant [direct effect = 0.445, SE = 0.02, 95% CI = (0.40, 0.49)]. More importantly, perceived entrepreneurial support partially mediated the relationship between proactive personality and entrepreneurial intention [indirect effect = 0.397, SE = 0.03, 95% CI = (0.34, 0.45)]. Mediation accounted for 47.1% of the total effect. Therefore, hypothesis 2 is supported.

**TABLE 3:** The mediating effect test.

Predictors	Model 1 (EI)			Model 2 (PES)			Model 3 (EI)		
	β	*t*	95%CI	β	*t*	95%CI	β	*t*	95%CI
PP	0.841	41.97***	[0.80, 0.88]	0.788	35.88***	[0.74, 0.83]	0.445	19.54***	[0.40, 0.49]
PES							0.503	25.71***	[0.47, 0.54]
*R* ^2^		0.54			0.46			0.68	
*F*		1761.74***			1287.56***			1595.49***	

N = 1,515. Bootstrap sample size = 5,000.

*p < 0.05, **p < 0.01, ***p < 0.001.

PP, proactive personality; PES, perceived entrepreneurial support; EI, entrepreneurial intention.

### Testing for moderated mediation

We used the PROCESS macro for SPSS (Model 59) to examine hypotheses 3, 4, and 5. Table 4 shows that the interaction between proactive personality and gender significantly predicts entrepreneurial intention (β = –0.111, *p* < 0.05), supporting hypothesis 3. However, the prediction for perceived entrepreneurial support was not significant (β = 0.065, *p* > 0.05); thus, hypothesis 4 was not supported. In addition, the interaction between perceived entrepreneurial support and gender significantly predicted entrepreneurial intention (β = 0.099, *p* < 0.05), supporting hypothesis 5.

**TABLE 4:** The moderated mediation effect test.

Predictors	Model 1 (PES)			Model 2 (EI)		
	β	*t*	95%CI	β	*t*	95%CI
PP	0.757	25.09***	[0.70, 0.82]	0.511	15.81***	[0.45, 0.57]
G	–0.410	−2.28*	[–0.76, –0.06]	–0.044	–0.31	[–0.32, 0.23]
PP × G	0.065	1.48	[–0.02, 0.15]	–0.111	−2.45*	[–0.20, –0.02]
PES				0.437	14.53***	[0.38, 0.50]
PES × G				0.099	2.50*	[0.02, 0.18]
*R* ^2^	0.467			0.684		
*F*	441.39***			654.16***		

N = 1,515. Bootstrap sample size = 5,000.

*p < 0.05, **p < 0.01, ***p < 0.001.

PP, proactive personality; PES, perceived entrepreneurial support; EI, entrepreneurial intention; G, gender. Gender: male = 0, female = 1.

**FIGURE 2:**
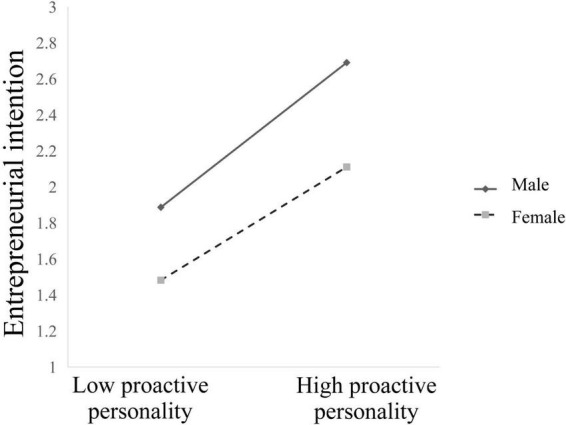
Gender moderated the relationship between proactive personality and entrepreneurial intention.

To better comprehend the essence of moderating effect of gender, we plotted the simple slopes. As presented in Figure 2, proactive personality was positively linked with entrepreneurial intention for male participants (β = 0.511, *t* = 15.81, *p* < 0.001), whereas the slope was weaker for female participants (β = 0.399, *t* = 12.48, *p* < 0.001).

**FIGURE 3:**
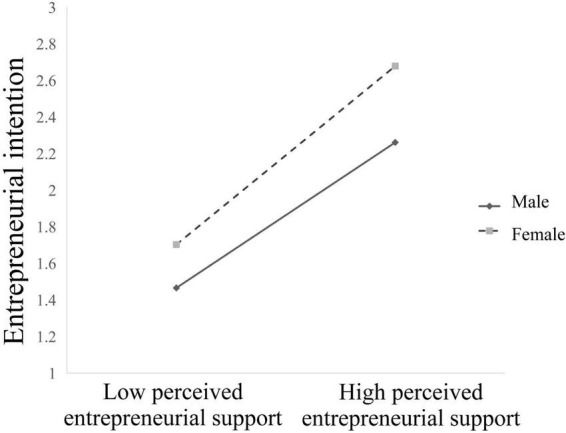
Gender moderated the relationship between perceived entreprenieurial support and entreprenieurial intention.

Figure 3 shows that perceived entrepreneurial support is positively associated with entrepreneurial intention for female participants (β = 0.536, *t* = 20.84, *p* < 0.001), whereas the association is weaker for male participants (β = 0.437, *t* = 14.53, *p* < 0.001).

In addition, we tested the conditional indirect effects of proactive personality on entrepreneurial intention through perceived entrepreneurial support. For male participants, there was a significant indirect effect of perceived entrepreneurial support, with an effect size of 0.331 [SE = 0.04, 95% CI = (0.25, 0.42)], while in female participants, the indirect effect was also significant and stronger, with an effect size of 0.441 [SE = 0.04, 95% CI = (0.38, 0.51)].

## Conclusion and discussion

This study proposes a moderated mediation model to understand the mechanisms underlying the relationships between proactive personality and entrepreneurial intention in Chinese private college students. It provides three theoretical contributions to research on proactive personality and entrepreneurial intention. First, the results of Table 2 and the model in Figure 2 show that the effect of proactive personality on entrepreneurial intention was significantly positive and acts directly in Chinese private college students. Second, perceived entrepreneurial support partially mediates the relationship between proactive personality and entrepreneurial intention. Thus, perceived entrepreneurial support can promote entrepreneurial intention for those who are proactive. Accordingly, if college students have a proactive personality and take full use of perceived entrepreneurial support, it will eventually lead to strong entrepreneurial intention. Third, this study indicates that the relationship between perceived entrepreneurial support and entrepreneurial intention and that between proactive personality and entrepreneurial intention are moderated by gender. The results remind us that improving the entrepreneurial intention of proactive individuals through perceived entrepreneurial support should consider gender differences, which only by perceiving sufficient entrepreneurial support and possessing a proactive personality can female students be willing to start a business. It can provide a strong theoretical basis and make such studies more inclusive from the perspective of Chinese private colleges that have strong entrepreneurial needs but may encounter more entrepreneurial difficulties in the current entrepreneurial environment.

### Impact of proactive personality on entrepreneurial intention

This study concludes that among Chinese private colleges, a higher proactive personality is related to a stronger intention to start a business. This finding supports hypothesis 1 and is congruent with previous findings that a proactive personality can further entrepreneurial intention (Mustafa et al., 2016; Munir et al., 2019). Only by establishing entrepreneurial intention can entrepreneurial education translate into entrepreneurial action. Individuals with high levels of proactive personality experience low levels of difficulty in career decision-making and feel higher confidence in entrepreneurship successfully (He et al., 2021). In addition, gender differences in the relationship between proactive personality and entrepreneurial intention were observed. This study indicates that proactive male students have stronger entrepreneurial intentions than proactive female students. Therefore, much attention should be paid to proactive personality as an antecedent factor of entrepreneurial intention, particularly for male students.

### Mediating role of perceived entrepreneurial support

The finding of this study demonstrates that perceived entrepreneurial support played a partial mediating role in the association between proactive personality and entrepreneurial intention (hypothesis 2). Based on the Huang et al.’s (2020) finding that the direct effect of entrepreneurial support on entrepreneurial tendency is significant and regulated by personal characteristics, He et al. (2021) indicated that perceived social support moderated the relationship between proactive personality and college students’ career decision-making difficulties. A previous study also indicated that the willingness of college students to engage in entrepreneurship was largely determined by combined effects of internal and external factors (Yi, 2021). This study further reveals the underlying mechanisms of proactive personality, perceived entrepreneurial support, and entrepreneurial intention. Our findings also indicate that perceived entrepreneurial support is a key mediating factor, implying that favorable entrepreneurial support is a key factor in fostering college students’ entrepreneurial intentions. Therefore, it is necessary for universities to provide entrepreneurial coaching and support for proactive students.

### Moderating role of gender

This study finds that the relationship between proactive personality and entrepreneurial intention is moderated by gender, supporting hypothesis 3. This study confirms a close relationship between proactive personality and entrepreneurial intention for both males and females and that the relationship between proactive personality and entrepreneurial intention is more significant in women than in men in Chinese private colleges. This may be because gender differences in personality traits, such as risk-taking tendency, sense of achievement for men, and job security for women, impacted entrepreneurial intention. Our findings are consistent with previous studies showing that the interaction between gender and entrepreneurial intention depends heavily on subjective social norms (Yang, 2013; Villanueva-Flores et al., 2021).

Gender moderates the relationship between proactive personality and entrepreneurial intention is proved in this study. Previous studies reached the general agreement that gender played a key role of determining the entrepreneurial intention (Yang, 2013; Villanueva-Flores et al., 2021). The interaction between gender and entrepreneurial intention heavily depended on the subjective social norm and showed great differences between developed countries and developing countries. On the one hand, female in developing countries is confined by more widespread gender stereotype and familial responsibilities, and on the other hand, the gender differences in personality traits (i.e., risk-taking tendency, sense of achievement for male, and job security for female) are also the key factors which influence entrepreneurial intention. This study confirmed that there is a close relationship between proactive personality and entrepreneurial intention for both male and female; the mediation effect is not significant for female compared to male. Women in developing countries were confined by more widespread gender stereotypes and familial responsibilities than those in the developed countries and thus had low levels of entrepreneurial intentions (Trivedi, 2017).

This study revealed that gender moderated the relationship between perceived entrepreneurial support and entrepreneurial intention, thus supporting hypothesis 5. Specifically, women have stronger entrepreneurial intention than men when they perceive positive entrepreneurial support. The finding validated that proactive personality and university support factors are the significant predictors of entrepreneurial intentions of female students (Sidratulmunthah et al., 2018). Regarding entrepreneurial support, although Rahaman et al. (2020) claimed that social network and business information would have more influence on male students’ entrepreneurial intention, Abbas (2015) asserted that gender moderates the influences of variables, such as proactive personality and perceived entrepreneurial support on college students’ entrepreneurial intention. This study indicated that proactive personality significantly influenced entrepreneurial intention in men and in women; however, the influence was less significantly in women than in men in Chinese private colleges. The possible reasons could be that men tend to be independent, whereas women’s entrepreneurial will be confined by the social norm, which is the foundation of entrepreneurial support. Villanueva-Flores et al. (2021) stated that informal social support had a key role in women’s entrepreneurial intention. Thus, female students need a supportive environment when deciding to start a business. As a result, when female students decide to establish a business, they require a supportive environment.

## Practical implications

From a practical standpoint, this study has direct implications for aspiring entrepreneurs, public policymakers, and entrepreneurship educators, with a particular focus on Chinese private colleges’ efforts to cultivate and foster students’ entrepreneurial intentions.

First, the effect of proactive personality on entrepreneurial intention is proven to promote college students’ entrepreneurial intentions and offers focused guidance. Given that individuals with proactive personalities are more likely to take action to influence their environments, educational institutions can help students develop proactive personalities through entrepreneurial instruction and practices. Entrepreneurship research groups should be developed to investigate effective approaches to enhance college students’ proactive personalities.

Second, Chinese private colleges and the government can make more efforts to provide students with a favorable entrepreneurial environment. Students can use supportive entrepreneurial ecosystems as incubators to develop their ideas into actual start-up activities. When students’ entrepreneurial intentions are developed, they will receive entrepreneurial support to help them achieve their goals of starting a business. For example, the support of college administrators and policymakers can cooperatively create a layered, relationship-friendly environment for private college students, including start-up funding, professional counseling, and risk-mitigation measures.

Additionally, gender was suggested to attract more attention as an important variable in entrepreneurial education and guidance because the impact of proactive personality on entrepreneurial intention and that of perceived entrepreneurial intention were influenced by gender. Moreover, building a supportive entrepreneurial environment is critical in transforming the youth perception to boost their knowledge in entrepreneurship to engage in business enterprises, as entrepreneurial intention is a conscious and planned decision. This study reveals that making female students understand advantageous entrepreneurial resources could promote youth entrepreneurship empowerment. Furthermore, the results suggest that private colleges could provide a supportive entrepreneurial environment and targeted entrepreneurship guidance for students, particularly for female students with high levels of proactive personality.

## Limitations and future directions

This study has several limitations. First, as the first empirical study to investigate the relationship between proactive personalities, perceived entrepreneurial support, and entrepreneurial intention of Chinese private college students, this study was conducted specifically with Chinese private college students using random sampling; the inferences are restricted. Expansion of the scope of respondents by investigating different types of colleges would further increase the value of relevant studies. In addition, turning entrepreneurial intention into entrepreneurial behavior is a complicated agenda, and longitudinal investigation is required to further clarify the influence mechanism between proactive personality and entrepreneurial intention. Finally, although gender is an important moderating variable of entrepreneurial intention, how gender moderates proactive personality and entrepreneurial intention varies in different culture. How gender moderates the key variables (i.e., proactive personality) and entrepreneurial intention requires further examination in future studies to clarify the previous contradict findings.

## Data availability statement

The raw data supporting the conclusions of this article will be made available by the authors, without undue reservation.

## Ethics statement

The studies involving human participants were reviewed and approved by the Ethics Committee of Hangzhou Normal University, Jinghengyi School of Education. Written informed consent for participation was not required for this study in accordance with the national legislation and the institutional requirements.

## Author contributions

HZ, JT, MZ, and YW: conceptualization. HZ, JT, and MZ: data curation, funding acquisition, and investigation. MZ and YW: validation and methodology. JT, YW, and MZ: formal analysis, writing—original draft, and writing review and editing. HZ: project administration. All authors contributed to the article and approved the submitted version.
